# Exploration of using a wall-climbing robot system for indoor inspection in occupied buildings

**DOI:** 10.1038/s41598-024-64642-z

**Published:** 2024-06-14

**Authors:** Leyuan Ma, Timo Hartmann

**Affiliations:** Technische Universität Berlin, Gustav-Meyer-Allee 25, 13355 Berlin, Germany

**Keywords:** Civil engineering, Electrical and electronic engineering

## Abstract

Indoor inspection robots operating in occupied buildings need to minimize disturbance to occupants and access high areas of a room and cramped spaces obstructed by obstacles for higher inspection coverage. However, existing indoor inspection robots are still unable to meet these requirements. This paper aims to explore the feasibility of applying wall-climbing robots to address these requirements. To this end, we propose a small-sized wall-climbing robot prototype that can move on common indoor surfaces. We extend the proposed prototype to support thermographic inspection by integrating thermal imaging technology into it. Experiment results show that the proposed robot prototype can reach more wall and floor areas for inspection than previously developed indoor inspection robots. It has also been demonstrated that the reduced size and the wall-climbing ability allow the robot to largely avoid human activity areas, thereby reducing disturbance to occupants. This study represents the first attempt to introduce wall-climbing robots into the indoor inspection domain and provides the initial validation of their advantages over existing indoor inspection robots regarding improving inspection coverage and minimizing disturbance to occupants. The findings in this study can provide valuable insights for the future design, selection and application of robotic systems for indoor inspection tasks.

## Introduction

Regular inspection of existing buildings is essential to ensure that the building continues to meet its expected service requirements^1^. The traditional way of building inspection involves building inspectors carrying specialized equipment to collect data from both the exterior and interior of a building, which is labor-intensive, costly and inefficient^2^. In recent years, the development of mobile building inspection robots has received increasing attention as they have great potential to reduce reliance on human inspectors and increase inspection efficiency^3^. Overall, the design of robots for building interior inspection is more demanding than those intended for building exterior inspection. Specifically, outdoor inspection robots typically operate in large open spaces, where they encounter few obstacles and building occupants^4^. In contrast, indoor inspection robots need to operate in occupied rooms with diverse furniture and appliances, potentially with occupants present^4^. This requires the robot to be able to avoid its inspection range being obstructed by these objects as much as possible^5,6^ and to minimize disturbance to occupants^7^. Furthermore, the existence of rooms with high ceilings requires indoor inspection robots to be able to reach some higher places. However, existing indoor inspection robots are still far from meeting the above requirements.

**Figure 1:**
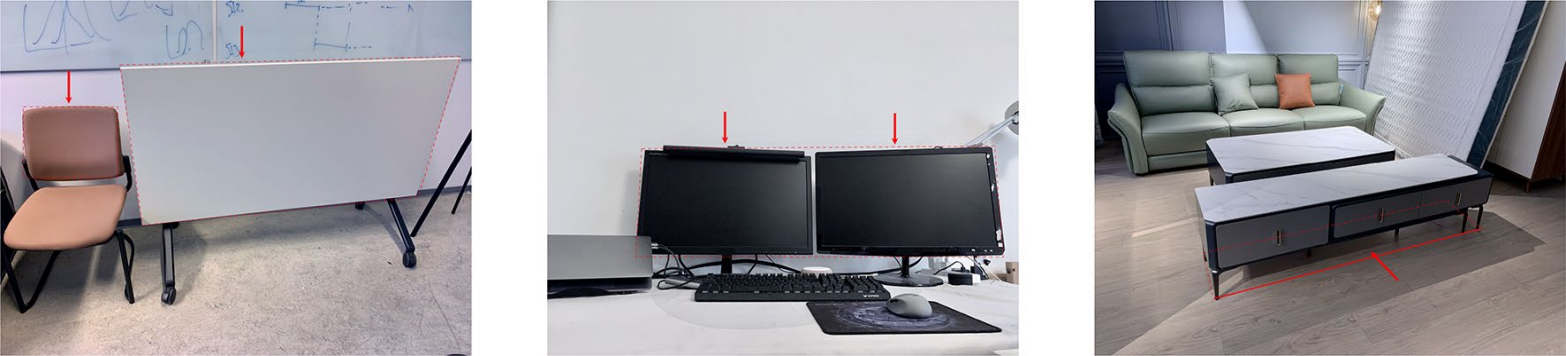
Examples of indoor scenarios. The chair, the table and the monitors shown in the figure have small gaps from the wall. The side table shown in the figure has a small gap from the floor. These small gaps are hard for existing indoor inspection robots to access, which causes the wall and floor areas obstructed by these objects unable to be inspected.

Indoor inspection robots that are commonly used today are unmanned ground vehicles (UGVs)^8,9^, which can only work from the floor. The lack of vertical mobility makes it hard to inspect high areas of a room^9,10^. Therefore, researchers have started to explore the use of unmanned aerial vehicles (UAVs) for indoor inspection. UAVs are superior to UGVs in terms of vertical maneuverability and they can fly into areas difficult for UGVs to reach^4^. However, in real-world indoor scenarios, some objects, as shown in Fig. 1, have small clearance from the floor or wall. Considering the size of the currently available UAVs (Table 1) and the need to maintain a safe distance from surrounding objects^11^, it is hard for UAVs to access narrow spaces behind or underneath the objects to inspect the wall or floor area obstructed by the objects^12^. Moreover, as mentioned earlier, indoor inspection robots should operate with minimal disturbance to occupants. However, as stated in^13^, robots approaching humans may elicit unpleasant and disturbing feelings, causing stress and anxiety; this feeling intensifies as the size and speed of the robot increase. UAVs flying indoors would cause more stress and anxiety for occupants than UGVs^13^.

**Table 1:** An overview of existing indoor inspection robots.

Indoor inspection robot	Robot type	Dimension	Source
Elios 3	UAV	480 mm (W) $\times$ 480 mm (L) $\times$ 380 mm (H)	Flyability
Asio	UAV	395 mm (Diameter)	Dronevolt
Lumicopter	UAV	426 mm (W) $\times$ 375 mm (L) $\times$ 364 mm(H)	Lumicopter
Stereo 2	UAV	390 mm (Diameter)	Multinnov
Pelican	UAV	651 mm (W) $\times$ 651 mm (L) $\times$ 188 mm(H)	^14^
THROO	UGV	420 mm (W) $\times$ 300 mm (L) $\times$ 140 mm (H)	^8^
Irma3D	UGV	520 mm (W) $\times$ 580 mm (L) $\times$ 315 mm (H)	^9^
Quicabot	UGV	625 mm (W) $\times$ 460 mm (L) $\times$ 410 mm (H)	^15^
Spot	UGV	500 mm (W) $\times$ 1100 mm (L) $\times$ 520 mm (H)	Bostondynamics

Given the above-mentioned limitations of existing indoor inspection robots, there is a need to explore the use of new types of robots to further improve inspection coverage and reduce intrusiveness to occupants. Wall-climbing robots are another type of robot that can overcome gravity constraints and move in a vertical direction. A small-sized wall-climbing robot can move over various surfaces and inspect the surfaces it traverses without occupying much space. Additionally, wall-climbing robots rely on walls for their vertical movement, allowing them to stay away from human activity areas^16^. Therefore, a small-sized wall-climbing robot has the potential to access more narrow spaces around the inspection target and reduce disturbance to occupants. However, existing publications have mainly developed wall-climbing robot systems to perform tasks on building exteriors^17–19^. To the best of our knowledge, no studies have yet validated the advantages of wall-climbing robots in enhancing indoor inspection coverage while minimizing disruption to occupants. Additionally, wall-climbing robots proposed in previous studies are still in the prototype development stage. There are no readily available wall-climbing robots that can be used to validate their advantages in indoor inspection. Therefore, to conduct this validation, this paper introduces a small wall-climbing robot prototype and subsequently assesses its suitability for indoor inspection and its capacity to access more areas compared to existing indoor detection robots in nine occupied rooms.

## Related work

### State of the art in robotic indoor inspection research

The previously designed indoor inspection robots can be categorized into unmanned ground vehicles (UGVs) and unmanned aerial vehicles (UAVs). Examples of indoor inspection UGVs are QuicaBot^15^ and two hybrid inspection robots proposed by Rea and Ottaviano^8,20^. QuicaBot^15^ is developed for post-construction quality assessment. The pan-tilt mechanism allows for enlarging the measurement range of the camera on the robot^15^. However, the robot was only tested in rooms of normal size. Its inspection coverage for rooms with high ceilings is still a concern. Furthermore, as it is designed for the inspection of newly constructed buildings, the design did not consider the need to avoid obstruction by furniture and other indoor objects. The hybrid inspection robots use a hybrid structure of tracks and legs to improve flexibility in overpassing obstacles^8,20^. However, like QuicaBot, they also have a limited vertical inspection range. In addition, they have only been tested in laboratory environments, where no objects obstructed the inspection targets. Their inspection coverage of occupied rooms remains to be tested. UAVs are more agile and have better views than UGVs^4^. For example, Gao et al.^21^ proposed a UAV-based explore-then-exploit system consisting of two UAVs for autonomous indoor facility inspection. The test of the proposed system shows that the UAVs can fly in confined spaces with a width of 0.8 m and collect data in any position and at any angle^21^. This guarantees the inspection coverage of target facilities in a cluttered environment where the distance between the obstacle and the facility is greater than 0.8 m^21^. However, the inspection range is reduced when the distance between the obstacle and the facility in the environment is less than 0.8 m. Moreover, as mentioned earlier, UAVs flying indoors can cause more unease for occupants than UGVs^13^.

To improve the inspection coverage of indoor UGVs and UAVs, a number of studies in recent years have developed methods to support inspection mission planning, such as data collection location planning and motion planning^9,21–25^. For example, Song et al.^24^ proposed a scan and flight planning method for LiDAR-carrying UAVs. The scan planning is based on a greedy algorithm that iteratively generates waypoints with optimized coverage. The path planning method uses the genetic algorithm to obtain the optimal visit order for the set of waypoints. The simulation demonstrates that the planned trajectory allows the UAV to achieve 91.67$\%$ scan coverage on the inspection target. However, in real-world scenarios (Fig. 1), the planned data collection locations may have obstacles nearby that impede the robot’s access. To guarantee high inspection coverage in such scenarios, applying a new type of robot that requires less workspace might be a solution.

A small-sized wall-climbing robot has great potential to meet this requirement. However, there are currently no such wall-climbing robots that can be used to validate its benefits in indoor inspection. Therefore, there is a need to develop an indoor wall-climbing robot for this validation. Choosing the appropriate attachment and locomotion method is the key to designing such a wall-climbing robot. The next section presents a review of existing adhesion and locomotion technologies and analyzes the applicability of these techniques to the design of the indoor wall-climbing robot.

### Existing adhesion and locomotion technologies for wall-climbing robots

The main adhesion mechanisms proposed in previous studies can be classified into magnetic adhesion^26^, bio-inspired adhesion^27–29^, pneumatic adhesion^18,30–36^, and electrostatic adhesion^37,38^. Since magnetic adhesion only works on ferromagnetic surfaces, it is unsuitable for most indoor surfaces^39^. Therefore, the following analysis focuses on the other three adhesion mechanisms and the locomotion mechanisms that commonly work with them.

Bio-inspired adhesion draws inspiration from the natural adhesive mechanisms of some organisms^27^, including geckos, insects and some vertebrates. Synthetic fibrillar dry adhesives resembling geckos’ setae have played an important role in the development of wall-climbing robots. Before the availability of mature synthetic fibrillar dry adhesives, researchers used flat elastomer adhesives as substitutes^28,40,41^. For example, Seo et al.^41^ developed a tank-like climbing robot using the flat dry elastomer proposed by Unver and Sitti^40^ as the material for the robot tracks. However, Unver and Sitti^40^ suggested that flat elastomers should be utilized on relatively smooth surfaces such as acrylic or glass with submicrometer-scale surface roughness amplitudes. The experiment conducted by Seo et al.^41^ also only demonstrated the robot’s ability to climb on acrylic surfaces. In addition, the effectiveness of such dry adhesives is highly sensitive to contamination^28,37,40,41^. When the adhesive gathers dust or other contaminants, it loses its adhesive force^28,40,41^. With the advent of gecko-inspired dry fibrillar adhesives, researchers have started to apply fibrillar adhesives in the design of wall-climbing robots. For example, Waalbot II^29^ implements the gecko-inspired fibrillar adhesives fabricated by Aksak et al.^27^ as the material for footpads. The fibrillar adhesives consist of arrays of fibers at the micro- and nano-scale. Murphy et al.^29^ and Aksak et al.^27^ have demonstrated that fibrillar adhesives can be adapted to rougher surfaces than flat elastomers because the effective contact area of fibrillar adhesives on rough surfaces is larger. However, similar to the dry flat elastomer, the main disadvantage of the dry fibrillar footpad is also the susceptibility to contamination^27^. Self-cleaning fibrillar adhesives are still under development^42^. Besides gecko-inspired fibrillar adhesives, interlocking mechanisms that mimic the structure of animal claws or spines are another example of bio-inspired attachment methods^43,44^. Daltorio et al.^43^ have tested the climbing performance of the Mini-Whegs^TM^ robot equipped with claw and spine mechanisms. The performance of claws and spines is independent of surface contaminants or dust. However, the experiment results in^43^ show that the types of surfaces the robot can climb on are limited to soft surfaces that the claws and spines can penetrate and porous hard surfaces with many surface asperities that claws and spines can hook onto.

Pneumatic adhesion typically leverages air pressure to generate suction force. Vacuum suction and vortex suction are two common methods within this category. Vacuum suction often employs suction cups as contact points between the robot and the surface. Depending on how the vacuum is generated, suction cups applied in existing wall-climbing robots can be further classified into passive^18,30^ and active suction cups^31–33^. A passive suction cup relies on the physical pressure exerted on the cup to expel the air between the cup and the contact surface^45,46^. Robots using such mechanisms do not need additional equipment and energy to maintain adhesion and therefore can be designed to be small and compact^30^. However, the performance of a passive suction cup is very susceptible to the conditions of its contact surface such as roughness, porosity, and cleanliness^30^. Any obstacles, dirt, dust or other contaminants on the surface can affect the seal of the suction cup, allowing air to enter the cup, leading to reduced adhesion. An active suction cup relies on an external mechanism such as a vacuum pump to remove air between the cup and the contact surface^45^. Compared to passive suction cups, active suction cups generate more suction force, allowing the robot to have a greater load capacity^46^. However, the robot using active suction is heavier and larger due to the need for additional components such as an external power supply, pumps, and hoses^31,32^. In addition, the length of external connections such as the hose connecting to the vacuum pump can restrict the robot’s working range^33,47^. External connections also limit the robot’s mobility, especially in environments with many obstacles, as long hoses or cables may become entangled in the obstacles^47^. Moreover, robots using suction cups for attachment typically employ leg-based or track-based locomotion mechanisms, where robot feet or caterpillar tracks are equipped with suction cups. Leg-based locomotion is slower than track-based systems as it takes time for the feet to detach from and re-attach to the surface^48^. The track-based locomotion has limitations in maneuverability since the continuous track design restricts the robot’s ability to change directions quickly^39,48^.

Vortex suction is a new pneumatic adhesion method based on the Bernoulli principle^31,49^. This approach relies on a high-speed rotational airflow to create a low-pressure zone within a cylindrical chamber^34^. The main advantage of this method is a high tolerance for surface obstacles, imperfections or contaminants, as there is no direct contact between the suction device and the target surface^34,39^. Depending on the source of the airflow, existing vortex suction units can be divided into two types. The first type of vortex gripper consists of a cylindrical chamber and two tangential nozzles^34^. The external compressed air enters the chamber from the nozzles and forms a high-speed vortex flow along the inner face of the chamber, creating a negative pressure zone at the center^34^. This enables a high-powered energy supply and ensures a high payload capacity for the robot. However, the drawback of this air supply method is similar to the active suction cup, where the operating range of the robot is restricted by the length of the external air-supply pipe^47^. It is also unsuitable for use in spaces with many obstacles^47^. The other type of vortex unit uses a high-speed rotating motor to drive the blades within the chamber, generating a high-speed rotating airflow^35,36^. A battery on the robot can power such a device. Therefore, it is s possible to use this vortex unit to build a tether-free robot capable of adapting to cluttered environments. Wall-climbing robots utilizing vortex suction for attachment often incorporate wheels for locomotion^35,36^. The motors driving the wheels provide precise and easy control over the robot’s movement, allowing the robot to change speed and direction easily.

Electrostatic adhesion is another adhesion method that has been developed in recent years to allow robots to adapt to a wider range of surfaces^37,38^. When a charged electro-adhesive mechanism approaches the wall surface, opposite charges are generated on the wall surface due to electrostatic induction, creating an attractive force between them^37,38^. Robots utilizing this attachment mechanism often employ tracked locomotion for maximum adhesion force^37,38^. Bisht et al.^37^ have concluded that the electro-adhesive mechanism can attach to glass surfaces with low power consumption and its performance is not affected by dust^37^. However, the adhesion performance test of an electrode panel^38^ showed that the adhesion on drywall surfaces was only one-third of that on glass due to the higher roughness. Furthermore, the adhesive force generated by the electro-adhesive mechanism is relatively weak compared to the pneumatic adhesion^37,38^. Greater attraction requires larger electro-adhesive caterpillar tracks, which results in a larger robot size^37^.

### Applicability analysis of existing wall-climbing robot technologies for indoor inspection

As indicated in the above section, different adhesion and locomotion mechanisms have advantages and disadvantages. The types of wall surfaces to which these adhesion mechanisms can be adapted also vary. Table 2 summarizes the advantages and disadvantages of existing adhesion mechanisms and the types of surfaces they can accommodate.

To design a wall-climbing robot for indoor inspection, it is important to understand the surface texture of indoor walls and the environment the robot would encounter^7^. Common types of interior wall finishes include plaster, drywall, ceramic tile, wood paneling and brick or stone veneer^50^. Drywall can be finished with various textures, such as smooth, orange peel, knockdown and popcorn. Brick or stone veneer closely resembles the appearance of traditional brick or stone walls, retaining the irregular and uneven texture of the brick or stone. Considering these types of textures and the inevitable presence of dust on interior walls, vortex suction, which is independent of surface conditions, is the most suitable adhesion mechanism for indoor wall-climbing robots. Considering that indoor inspection robots need to navigate in rooms containing various obstacles such as furniture and appliances, the vortex gripper with external air-supply pipes is unsuitable because the external pipe restricts the robot’s range of motion and can become entangled in obstacles^47^. The other type of vortex gripper can avoid these problems, as it relies on the high-speed rotation of blades inside its chamber to generate suction. By combining this attachment mechanism with a wheel-driven motion mechanism, it is possible to obtain a tetherless robot that can move freely and adapt to various indoor surfaces.

**Table 2:** An overview of existing adhesion mechanisms.

Adhesion methods			Advantages	Disadvantages	Adaptable surface
Bio-inspired adhesion	Dry fibrillar adhesives		Energy saving	Contamination sensitive	Smooth, low-roughness surfaces
	Interlocking mechanisms		Energy saving, immune to contaminants	Possible damage to the wall	Soft, high-roughness surfaces
Pneumatic adhesion	Suction cups	Passive suction sups	Energy saving	Contamination- and obstacle-sensitive	Smooth surfaces
		Active suction cups	High payload capacity and attraction	Large size, limited operating range and mobility	Smooth, low-roughness surfaces
	Vortex suction	Vortex gripper with external air supply	High payload capacity and attraction	Large size, limited operating range and mobility	Smooth, rough surfaces
		Vortex gripper with blades	Immune to contaminants, applicable to diverse surfaces, high mobility	Energy costing	Smooth, rough surfaces
Electrostatic adhesion			Energy saving, immune to dust	Low adhesion force, low payload, large size	Smooth, low-roughness surfaces

### Research motivation

For robot-assisted indoor inspection, it is important to achieve high coverage of occupied rooms while minimizing disturbance to occupants. Previous studies have applied UAVs to complement the limited vertical inspection range of UGVs and proposed inspection mission planning methods to determine optimal data collection locations and paths to visit each identified location. However, in the real world, there are still some data collection locations that are inaccessible to existing inspection UAVs because of the large workspace they require. Furthermore, indoor inspection UAVs still have the problem of causing unease or discomfort to occupants. This has motivated us to explore the use of a small-sized wall-climbing robot to solve the above problems.

Since the development of wall-climbing robots is still an emerging field, most of them are still in the prototyping stage and have not yet reached the market. In addition, the main application scenarios currently considered for the development of wall-climbing building inspection robots are limited to building exterior inspection^18,31,35,49,51^. Therefore, no readily available wall-climbing robots can be used to validate their ability to solve the aforementioned problems of existing indoor inspection robots. To validate the ability of wall-climbing robots to improve indoor inspection coverage and reduce disturbance to occupants, there is a need to develop an indoor wall-climbing robot prototype. Therefore, the main aim of this study is to develop a wall-climbing robot prototype that can be used for indoor inspection and validate its advantages over existing indoor inspection robots in terms of inspection coverage and less disturbance to occupants.

The review of existing wall-climbing robot technologies suggests that a vortex suction device that relies on the high-speed rotation of blades to generate negative pressure and a wheel-driven locomotion mechanism are most suitable for constructing an indoor wall-climbing robot. Current wall-climbing robot prototypes using such mechanisms are primarily designed for outdoor applications^35,36^, where robots rarely encounter confined spaces. Consequently, the hardware size of the robot is not a major consideration in these designs. To the best of our knowledge, no research has been conducted to apply the above mechanisms to construct a small-sized wall-climbing robot to access narrow indoor spaces for inspection. Therefore, we applied the above mechanisms in the development of the wall-climbing robot prototype for indoor inspection, where we carefully considered the size constraints imposed by tight spaces that exist indoors. The next section presents the detailed architecture of the proposed indoor wall-climbing robot system.

## The architecture of the indoor wall-climbing inspection robot system

### Mechanical structure of the indoor wall-climbing robot platform

An Electric Ducted Fan (EDF) is utilized as the adhesion mechanism of the proposed wall-climbing robot prototype. The EDF consists of a brushless DC motor (BLDC) and an impeller encased by a cylindrical duct and a shroud^52^. Since no external equipment has to be attached, this option allows for an untethered design solution. The EDF used in this prototype is one of the QX-MOTOR ducting set series. The specification of the EDF is shown in Table 3. The locomotion mechanism of the robot includes four DC geared motors that drive the four wheels of the robot, and each driving wheel can generate a driving force^36^. If different rotation speeds are given to the right and the left motors, the robot will change its direction. The motor used in the robot prototype is the N20 micro geared DC motor with a size of 35.2 mm $\times$ 12 mm $\times$ 10 mm. The wheel customized for this motor has a diameter of 43 mm. Wheels with such a small diameter reduce the distance between the wall and the robot base, enabling a high suction efficiency. Detailed specifications of the N20 motor and the wheel are included in Table 3.

**Table 3:** Specifications of the indoor wall-climbing robot components.

Component	Specification	
N20 micro geared DC motor	Motor model	GA12-N20
	Nominal voltage	6 V
	No load speed	100 rpm
	Stall current	200 mA
	Stall torque	3.2 kg-cm
	Size	35.2 mm (L) $\times$ 12 mm (W) $\times$ 10 mm (H)
	Weight	9 g
Wheel	Diameter	43 mm
	Width	19 mm
	Weight	18 g
Electric Ducted Fan (EDF)	Motor model	QF2611 4600 Kv
	Revolution speed	4600 rpm/v
	Inner Diameter	54 mm
	Shroud Diameter	63 mm
	Blades Diameter	50 mm
	Maximum Voltage	14.8 V(4S)
	Maximum Amps	32 A
	Maximum Thrust	780 g
	Weight	76 g

The chassis of the robot (190 mm $\times$ 190 mm $\times$ 2.5 mm) was 3D printed with Polylactic Acid which allows for a lightweight structure while offering enough strength. The dimensions of the chassis and the holes on it were customized according to the size of the selected components to be placed on the robot base. Since the weight of the wall-climbing platform is a dominating design factor, which greatly affects the robot’s adhesion performance^53^, a number of long holes have been made to reduce the weight of the chassis. The weight of the printed plate is 82 g.

**Figure 2:**
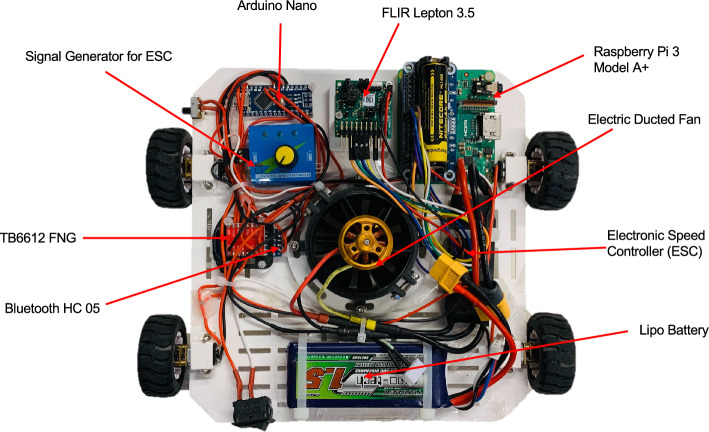
The indoor wall-climbing robot prototype. The electric ducted fan is the main adhesion mechanism for the prototype. Electronic Speed Controller (ESC) is used to control the speed of the rotation speed of the ducted fan. Arduino Nano is programmed to control the movement of the robots. The robot is supplied by a LiPo battery that has three cells and 1500 mAh capacity.

The clearance height between the suction unit and the wall should be minimized to ensure a high suction force. However, although indoor surfaces are relatively flat, small bulges can exist on some decorated walls^39^. This means that it is better to have enough open space under the suction unit to enable the robot to pass over such obstacles, which, however, causes a loss of suction force. According to the study of^36^, a soft skirt installed under the suction unit can not only improve the attraction efficiency but also guarantee the robot’s obstacle-surmounting ability as it can easily deform when it hits a bump. Thus, a circle of 6 mm-height soft skirt made of foam rubber is added at the bottom of the robot chassis. The clearance height between the suction unit and the wall is reduced to 8 mm. Figure 2 shows the final prototype of the proposed indoor wall-climbing robot.

### Mechatronic scheme of the indoor wall-climbing robot platform

**Figure 3:**
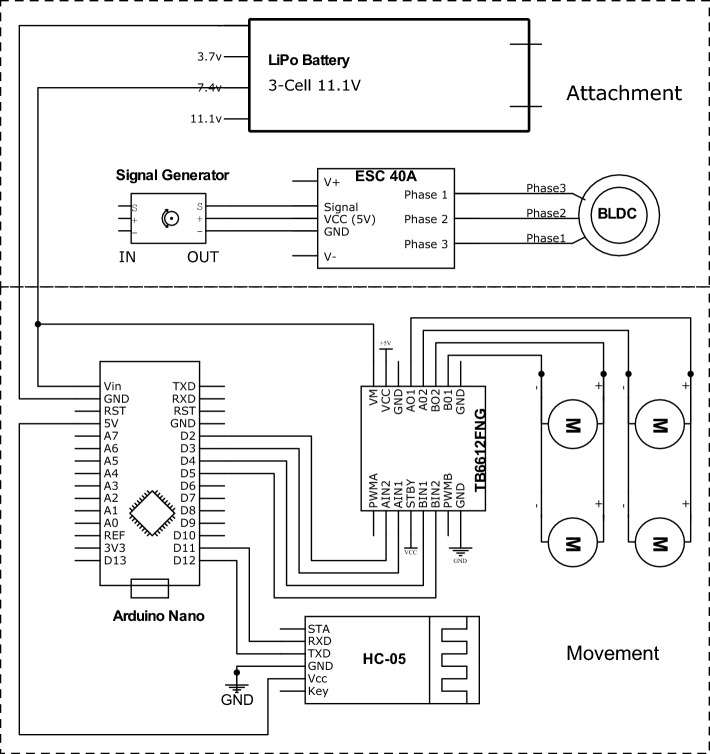
Circuit diagram of indoor wall-climbing robot platform.

The objective of the mechatronic design of the wall-climbing robot is to systematically combine the mechanical and electronic components and to ensure stable attachment and continuous movement. For the robot to adhere to the surface, the rotation speed of the brushless DC motor (BLDC) of the EDF should be enough to generate sufficient attraction force. To control the speed of the BLDC, an Electronic Speed Controller (ESC) and a signal generator that sends signals to the ESC are applied. According to the specification of the EDF in Table 3, the selected ESC is called Hobbywing Skywalker ESC-40 A, which has a continuous current capability of 40 A. The top part of Fig. 3 shows their connection.

The movement control part comprises a microcontroller board Arduino Nano, an HC-05 Bluetooth module, an Android Bluetooth control application, and a TB6612 FNG motor driver. Arduino Nano is the small-sized version of the more common Arduino Uno board. It is based on the same Atmel ATmega328P microprocessor as Arduino Uno, which supports UART TTL (5 V) serial communication^54^. HC-05 is a Bluetooth SPP (Serial Port Protocol) module designed for wireless serial connection setup^55^. TB6612 FNG is an H-Bridge motor driver that permits simultaneous control of the speed and the direction of a DC motor. Compared to the commonly used L298N motor driver, TB6612 FNG has the advantage of small size and low heat dissipation, making it ideal for usage on the indoor wall-climbing robot platform.

In this work, the Android Bluetooth Controller App created by^55^ is installed on an Android cellphone. An HC-05 module serves as a medium for communication between Arduino and the Bluetooth Controller App. When the predefined key on the app is pressed, the corresponding data is sent to HC-05. Then, HC-05 operates as a transmitter, sending the received data to the Arduino board. On the Arduino board, commands controlling the direction and the speed of the motor are activated and control signals are sent to the TB6612 FNG motor driver. In this way, the smartphone can wirelessly control the robot’s motion. The motion control program which corresponds to the data configured in the Bluetooth Controller App is written and uploaded to the Arduino Nano board in the Arduino integrated development environment (IDE). The lower half part of Fig. 3 shows the circuit diagram of the movement control.

The whole wall-climbing robot platform is supplied by a portable LiPo battery that has three cells and 1500 mAh capacity. The battery enables the robot to operate for around ten minutes. It is directly attached to the robot platform, avoiding the use of an external power supply cable. This makes the wall-climbing robot suitable to work in complex environments with a lot of obstacles. Table 4 illustrates the specification of the platform.

**Table 4:** Specifications of the indoor wall-climbing robot platform.

Parameter	Details	
Dimension (including wheels)	Width: 228 mm (190 mm + 19 mm $\times$ 2) Length: 190 mm Height: 110 mm	
Power supply	1500 mAh, 3S, 11.1 V Lithium Polymer battery	
Clearance height between the suction unit and the surface	8 mm	
Weight (including batteries)	538 g	
Maximum climbing speed	18 cm/s	

### The integration of inspection sensors into the wall-climbing robot platform for indoor inspection

To assess the robot’s data collection ability and coverage, the robot platform should be equipped with specific inspection sensors or equipment to obtain data collection ability. Infrared thermography is one of the most commonly used non-destructive testing technologies for indoor inspection^56,57^. It measures the infrared radiation density from different parts of a surface, converts it into temperature, and displays an image of the temperature distribution^58^. Defects, such as cracks, air leakage, moisture ingress and damaged insulation, would interrupt heat transfer through building elements such as walls and floors, causing abnormal temperature distributions on their surfaces^56^. Therefore, lots of defects can be detected by collecting and analyzing thermal images from building elements. We thus explored how to integrate thermal imaging into the proposed wall-climbing robot platform. The details are presented in the following subsections.

#### The selection of the thermal camera module

Important parameters to consider when choosing a thermal camera (also called infrared camera) include spectral range, spatial resolution, and thermal sensitivity^57^. Spectral range indicates the range of wavelengths that the infrared (IR) sensor in the camera is sensitive to^57^. It varies from the short-wave band (1.4-3 µm) to the long-wave band (8-15 µm)^59^. Short-wave IR (SWIR) cameras are suitable for high-temperature inspections, while long-wave IR (LWIR) cameras are more appropriate for observing objects at room temperature^58^. Spatial resolution relates to the number of pixels of the output thermal image, where more pixels mean a higher level of detail^57^. For indoor thermography, the IR camera is recommended to reach a minimum of 160 $\times$ 120 pixels^60^. Thermal sensitivity represents the smallest temperature difference the IR detector can distinguish^57^. The measurement of thermal sensitivity is noise equivalent temperature detection (NETD), which is typically described in milli-Kelvin (mK). It is recommended to use IR cameras with a NETD of at least 80 mK (0.08 ^∘^C) for indoor thermographic inspection^60^.

There are a variety of IR cameras on the market that meet the above requirements. However, the one to be carried by the proposed indoor wall-climbing robot is more demanding. It should not only be compact, lightweight and energy-saving but also be able to support wireless communication. Therefore, the FLIR’s LWIR camera module Lepton 3.5, which is the latest and smallest version of FLIR’s micro thermal camera (MTC) family^61^, is selected as the core for the thermographic inspection unit. This MTC core with a dimension of 12.7 mm $\times$ 10.50 mm $\times$ 7.14 mm is designed for easy integration into third-party products such as mobile devices and other electronics^62^. It produces thermal images with a resolution of 160 $\times$ 120 pixels and can detect temperature differences below 50 mK (0.050 ^∘^C). It weighs 0.9 grams and operates on a low power consumption of 150 mW^62^. These characteristics make it an ideal choice for employment in this project. In addition, its minimum focus distance is 10 cm, which means the closest distance at which the camera module can obtain a focused image is 10 cm^62^. Therefore, the lens of the camera module needs to be placed at least 10 cm from the bottom of the robot.

#### The control of the thermography unit

To get real-time thermal images, the FLIR Lepton thermal camera module is connected to a small single-board computer, Raspberry Pi 3 Model A+, via the FLIR Lepton Thermal Camera Breakout Board V2.0. The GPIO (General Purpose Input and Output) pins of the Raspberry Pi board are the principal manner of connecting to other electronic boards^63^. Some of them can be used as interfaces for embedded protocols such as I2C (Inter-Integrated Circuit) and SPI (Serial peripheral interface)^63^. The Lepton module transfers thermal data to the Raspberry Pi board over the SPI interface and uses the I2C protocol for accessing specific camera functions^64^.

**Figure 4:**
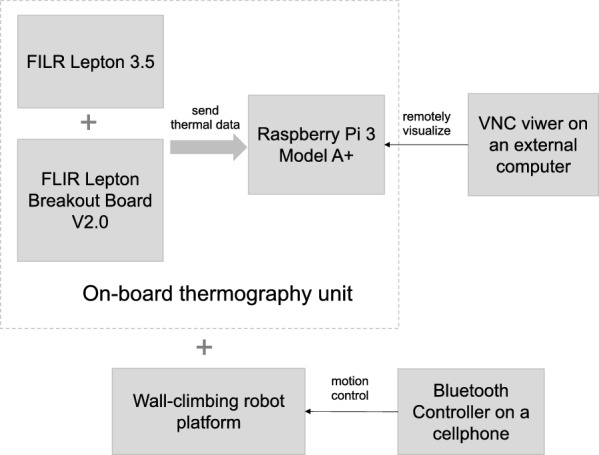
The wall-climbing robot-based thermographic inspection system. The FLIR Lepton thermal camera module is connected to Raspberry Pi 3 Model A+ via the FLIR Lepton Thermal Camera Breakout Board V2.0. The graphical desktop sharing system VNC (Virtual Network Computing) installed on an external computer remotely accesses the desktop of the Raspberry Pi and visualizes the collected thermograms.

Pure Engineering’s example code^65^, which creates a graphical user interface application for displaying real-time thermal outcomes, is executed on Raspberry Pi. The built-in Bluetooth and wireless LAN allow wireless control of the Raspberry Pi. To remotely access the desktop of the Raspberry Pi and visualize the collected thermograms, the graphical desktop sharing system VNC (Virtual Network Computing) is applied. The VNC viewer installed on an external computer can be connected to the VNC server on Raspberry Pi over a direct connection or a cloud connection. The direct connection requires that both devices are connected to the same local network and that the IP address of the Raspberry Pi is known. The cloud connection requires both sides to be logged into the same RealVNC account. The thermography unit including the thermal camera module and the control part weighs 92 g. The integration of the thermography unit and the wall-climbing robot platform is shown in Fig. 4.

## Experiments and results

### Testing the wall-climbing and thermal data collection capabilities of the robot

**Figure 5:**
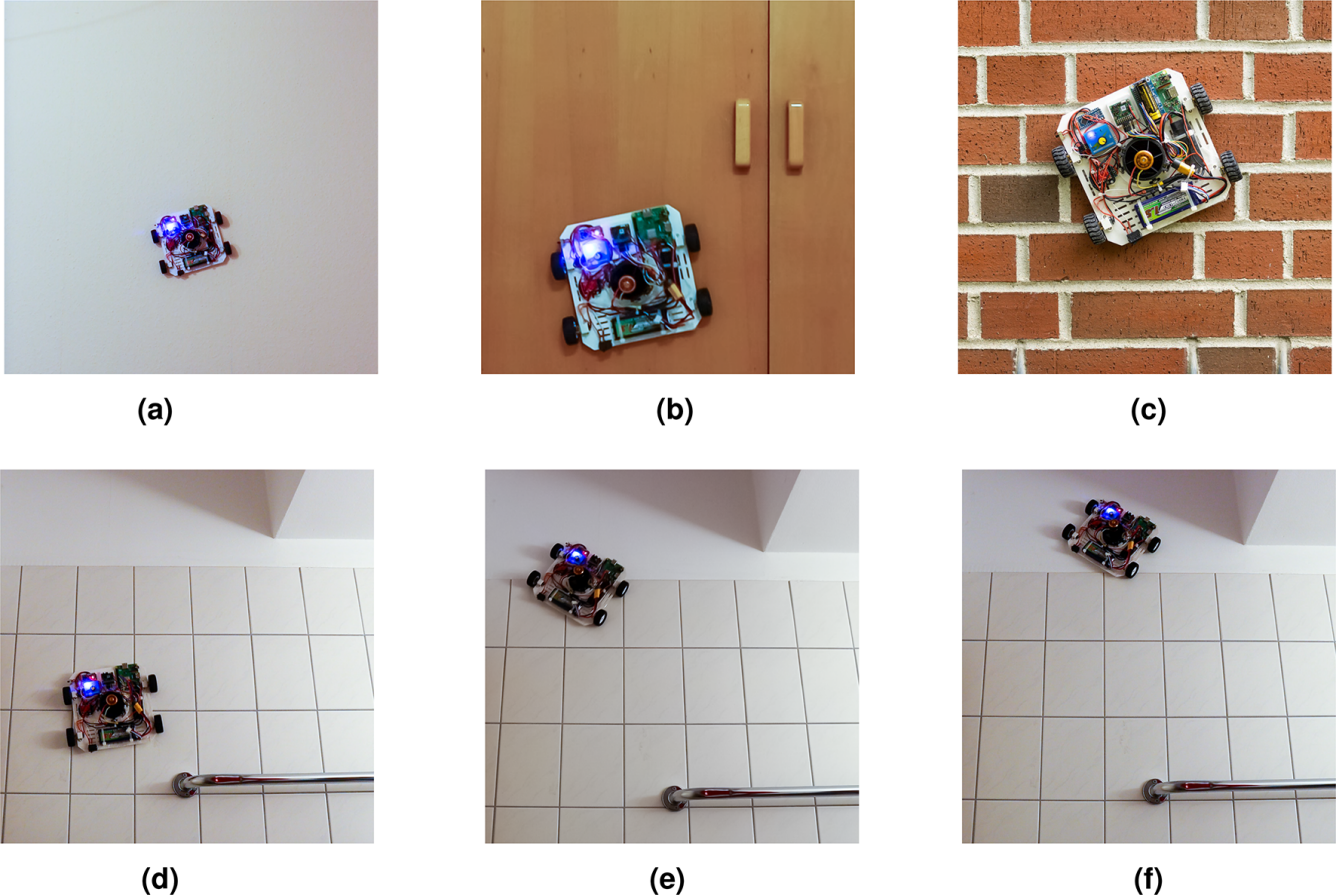
Snapshots of the proposed wall-climbing robot platform equipped with the thermography unit climbing up different surfaces. (**a**)–(**d**) show the proposed robot prototype climbing on drywall covered by ingrain wallpaper, a wooden panel, a brick veneer wall, and a wall covered by ceramic tiles.

The developed wall-climbing robot prototype equipped with the thermography unit was tested in a furnished apartment. The following four kinds of surfaces were selected to test the climbing ability: (1) drywall covered by ingrain wallpaper, (2) a wooden door, (3) a brick veneer wall, and (4) a wall covered by ceramic tiles. Before starting the robot, a digital battery capacity checker was used to check the battery level and ensure that the robot had sufficient power. After turning on the switch of the robot, the Bluetooth Controller app on a cellphone was paired with the HC-05 Bluetooth Module on the robot. Then, by slowly turning the knob on the signal generator, the rotating speed of the EDF was adjusted to an appropriate value enough to hold the robot against the wall. After completing the above preparations, we wirelessly controlled the wall-climbing platform using a cellphone. Figure 5 shows the snapshots of the proposed robot climbing up four types of surfaces. As shown in Fig. 5d–f, the robot can pass over surfaces of changing heights. It is also observed that the robot gets additional acceleration due to gravity when it moves downwards. The videos included in http://dx.doi.org/10.14279/depositonce-14989 demonstrate more details about the robot’s performance on the wall.

To visualize the thermal data collected by the robot, a direct connection was established between an external computer and the Raspberry Pi. After gaining remote access to the Raspberry Pi, the operator used the external computer to control the execution of the Pure Engineering’s example code on the Raspberry Pi. When the robot started to move and the code was run, the real-time thermal video collected by the FLIR Lepton thermal module was displayed on the computer.

**Figure 6:**
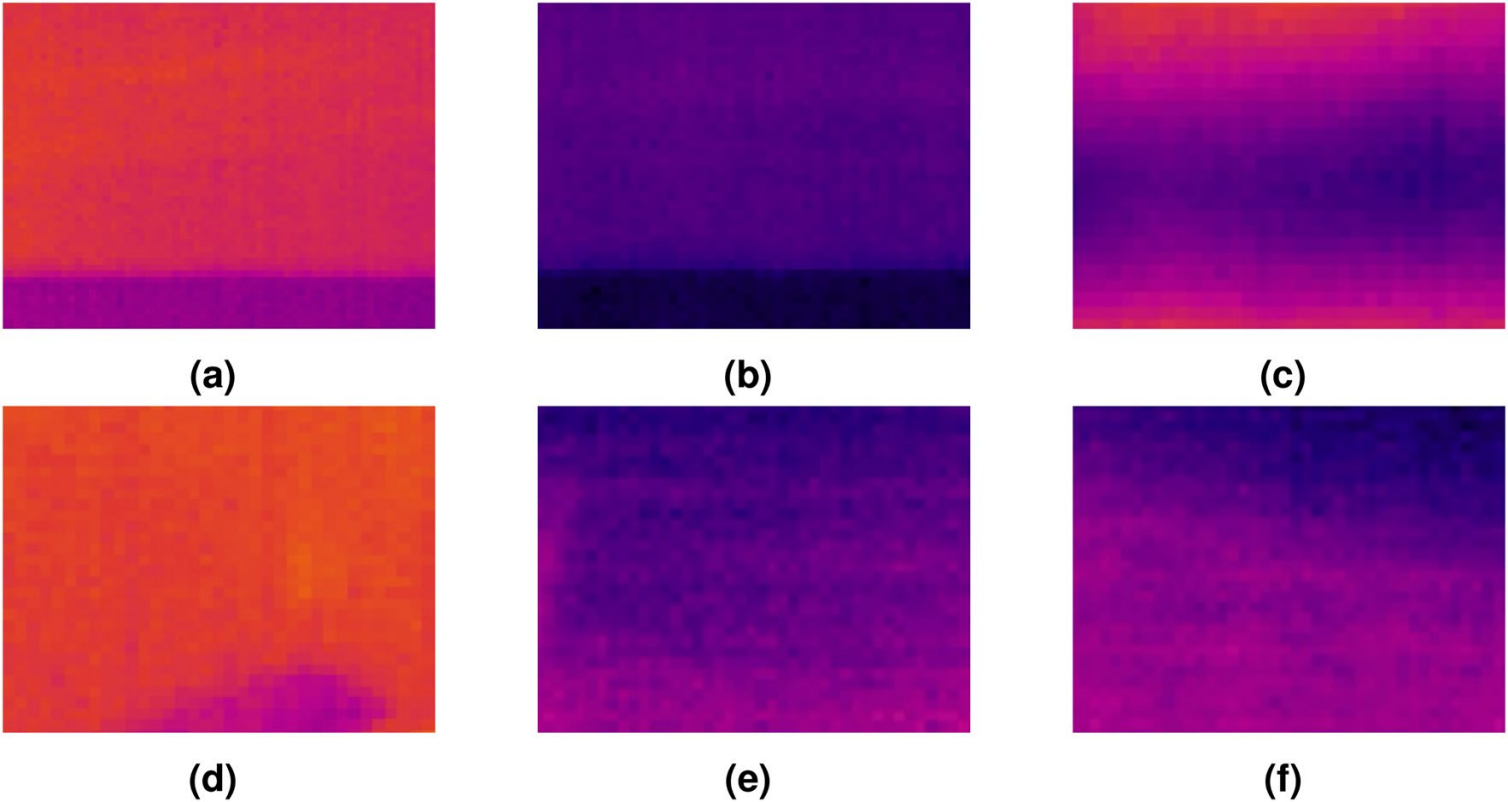
The output thermal images of the robotic thermography system. (**a**) and (**b**) may represent a hot water pipe and a cold water pipe, respectively. (**c**) and (**d**) probably show moisture problems. (**e**) and (**f**) may indicate an area of air leakage.

Ironbow thermal palette, a general-purpose palette using color to show heat distributions and subtle details, is applied to represent the thermal data obtained from the FLIR Lepton module^66^. Hot objects are shown in lighter and warm colors while colder objects are in dark and cool colors^66^. A set of snapshots of the thermal video is shown in Fig. 6. Distinct color differences are visible in each picture, indicating temperature changes in the surface area traversed by the robot. According to the ironbow palette defined by FLIR^66^, the following conclusions can be made: (1) the temperature of the object in Fig. 6a is higher than that in Fig. 6b; (2) the purple areas in Fig. 6c,d are colder than their surroundings; (3) the upper areas of Fig. 6e, f are colder than the lower areas. Therefore, Fig. 6a,b may represent a hot water pipe and a cold water pipe, respectively. Figure 6c,d probably show moisture problems. Figure 6e,f may indicate an area of air leakage.

### The comparison of the proposed wall-climbing robot prototype and the existing indoor inspection robots

**Figure 7:**
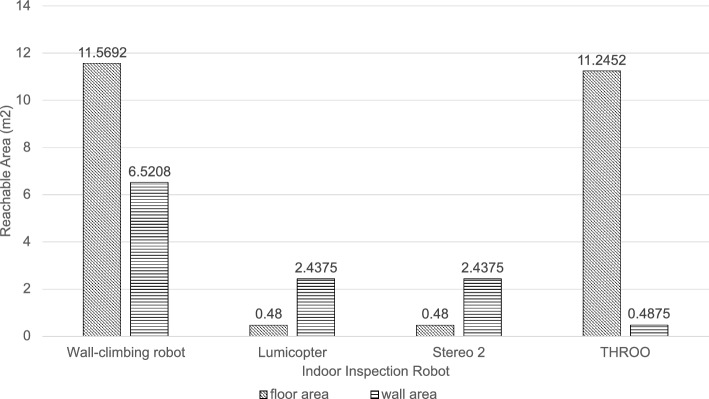
Comparison of the reachable area of existing robots and the proposed robot prototype. The area reachable by the proposed prototype is approximately five times that of the Lumicopter and Stereo 2, and 1.5 times that of the THROO.

**Figure 8:**
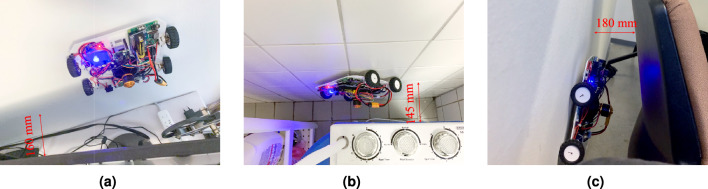
The proposed wall-climbing robot operating on surfaces that are inaccessible to existing robots. The gaps between the inspection targets and the obstacles shown in figure (**a**), (**b**), and (**c**) are 160 mm, 145 mm, and 180 mm, respectively. The proposed robot prototype can inspect these targets.

To compare the range of areas that can be reached by the proposed wall-climbing robot prototype with the existing indoor inspection robots, we selected three robots with relatively small sizes (Lumicopter, Stereo 2 and THROO) from the existing indoor inspection robots listed in Table 1. We investigated nine occupied rooms, including three office rooms, three living rooms and three bedrooms, and focused on objects that have small clearance from the wall or floor. We found 22 objects that have clearance between 30-500 mm from the wall or floor. The wall area behind these objects is 7.45 $m^2$ and the floor area under these objects is 11.83 $m^2$. By comparing these gaps with the dimension of each robot, whether the robot can access these spaces can be determined. Figure 7 shows the area of the region reachable by each of the four robots. Figure 8 presents three scenarios where the proposed robot prototype can reach places that other robots cannot.

Overall, as shown in Fig. 7, the area reachable by the proposed prototype is approximately five times that of the Lumicopter and Stereo 2, and 1.5 times that of the THROO. Specifially, the proposed wall-climbing robot prototype and the unmanned ground vehicle THROO can reach most of the floor area underneath the objects due to their low height. However, THROO can reach only about 4$\%$ of the wall area that the wall-climbing robot can reach because there is not enough space in front of the wall to accommodate THROO and THROO’s vertical mobility is limited. The UAVs Lumicopter and Stereo 2 can reach more wall areas than THROO due to their flying ability. The working space of the proposed wall-climbing robot is 228 mm (W) $\times$ 190 mm (L) $\times$ 110 mm (H), which is 1/12th of the working space required by Lumicopter and Stereo 2. This allows it to access more narrow spaces than Lumicopter and Stereo 2.

## Discussion

This study is conducted based on the assumption that it is possible to improve the inspection coverage of existing indoor inspection robots and reduce disturbance to occupants by applying a small-sized wall-climbing robot. To validate this assumption, we developed a small-sized wall-climbing robot prototype that occupies a workspace about 1/12th of that of existing robots. When applying the proposed robot prototype for wall inspection, its workspace is away from human activity space, causing less disturbance to humans. In addition, the proposed robot prototype can collect data from more wall areas and floor areas since it has enough vertical mobility and can access more narrow spaces around the floor and the wall. However, to understand exactly how much the proposed robot prototype improves inspection coverage, practical experiments need to be conducted in the future by placing previously developed indoor inspection robots and the proposed robot prototype in the same room to perform data collection. Moreover, further on-site research is needed to investigate residents’ attitudes towards the proposed wall-climbing robot prototype and other existing robots operating in their vicinity.

Although the experiments have demonstrated that the proposed wall-climbing robot prototype can collect data from more indoor areas than existing indoor inspection robots, there are still many areas covered by objects such as built-in furniture that are inaccessible to robots, which is a difficult problem to solve. Furthermore, the main purpose of this study is to validate the applicability of wall-climbing robots for indoor inspection and their ability to improve inspection coverage and reduce disturbance to occupants. Therefore, we developed a rudimentary wall-climbing robot prototype and conducted experiments to accomplish this validation. We have not conducted specific experiments to understand the performance of the robot, such as adhesion, speed, load capacity and working time. Nonetheless, the experiments that have been conducted have allowed us to identify some directions needed to improve the performance of the prototype.

First, the integration of thermal imaging technology into the wall-climbing robot allows the robot to collect thermal images from the surface it traverses. However, the area that can be inspected in one shot is very limited due to the fixed field of view of the camera and the small distance between the camera and the target surface. Consequently, the information included in a single image is often insufficient for an effective interpretation. Therefore, to allow wall-climbing robots to perform more effective visual inspection, image-stitching algorithms need to be applied.

Second, the proposed prototype is only able to climb along a single surface. Its ability to transfer between intersecting surfaces has not been realized yet. In reality, there are many scenarios where the robot needs to transition between different surfaces to reach specific locations. Changing the robot structure to a double-cavity structure with two vortex suction units that can switch between a sucking and a leaky chamber might be a solution.

Third, although the proposed wall-climbing robot prototype is better at accessing narrow spaces and avoiding disruptions to humans, it suffers from a smaller payload and shorter continuous working time. Therefore, using the wall-climbing robot alone is not sufficient to carry large inspection equipment and inspect large buildings. Thus, the wall-climbing inspection robot is not supposed to replace other robotic methods. Developing a multi-robot system to leverage the strengths of different types of robots is required in future research. For example, the ground-based inspection robot can conduct an initial and quick scan of the room and then request the assistance of the wall-climbing robot to inspect specified areas inaccessible from the ground. The ground robot can also serve as a support center, providing essential communication relays, computational capabilities, and on-demand recharging^67^.

Finally, future research should take measures to increase the operational time of the wall-climbing robot. The reason for the short operational time is that the high-speed rotation of the brushless motor consumes a significant amount of power to generate enough suction force. Therefore, the operational time can be increased by either using a battery with a higher capacity or reducing energy consumption. But it is worth noting that larger capacity batteries tend to be heavier, necessitating a greater suction force for adhesion. Therefore, an optimum battery capacity to weight ratio needs to be found. A structural topology optimization^47^ of the robot chassis could be performed to minimize the weight of the robot platform and reduce the energy consumption. Another method to reduce energy consumption is to improve the efficiency of the suction device. To achieve this, future research should consider replacing the commercial Electric Ducted Fan (EDF) employed in the existing design with an upgraded vortex unit. Different design variables regarding the shroud radius and the gap height between the EDF and the test surface need to be tested to find an optimal design solution for the vortex unit.

## Conclusion

Existing indoor inspection robots face challenges in reaching high places, accessing narrow spaces around inspection targets and avoiding human activity areas, resulting in limited inspection coverage and disruption to occupants. To validate the ability of wall-climbing robots to mitigate these problems, this paper proposes a small-sized indoor wall-climbing robot prototype and integrates thermal imaging technology into it. The size of the proposed prototype is about 1/12th of that of existing indoor inspection robots. Field tests of its wall-climbing and thermal imaging capabilities have been conducted. The results show that the wall-climbing robot-based thermography system can obtain real-time thermal data from the surface it traverses, and points of interest in the data can be identified by human operators. The experiments also demonstrated that the proposed robot prototype can reach more wall and floor areas for inspection than existing indoor inspection robots and that the wall-climbing ability allows the robot to effectively avoid human activity areas.

Despite the above advantages demonstrated in indoor inspection, the current robot prototype is still in its preliminary stage and has some limitations, including the inability to perform surface-to-surface transitions, low load capacity, and short continuous working time. To enable the robot to conduct surface-to-surface transitions, there is a need to change the robot structure to a double-cavity structure with two vortex suction units. To improve the load capacity of the robot and extend the operational time, the design of the electric ducted fan should be improved in future research to increase its suction efficiency. Furthermore, advanced design techniques, such as topology optimization, can help optimize the robot’s structural layout and minimize the robot’s weight, thus reducing the amount of energy required to attach the robot to the wall. Moreover, developing a multi-robot system that includes different types of robots is important to compensate for the limited load capacity and working time of wall-climbing robots.

## Data Availability

Data used in this study can be requested from the corresponding author.
